# The Efficacy and Safety of Mizoribine versus Mycophenolate Mofetil for the Treatment of Renal Transplantation: A Systematic Review and Meta-Analysis

**DOI:** 10.1155/2022/5717068

**Published:** 2022-07-22

**Authors:** Jie Chen, Hua Liu, Wenjun Yin, Zhengguang Xu, Zekai Chen, Wingkeung Yiu

**Affiliations:** ^1^Urinary Surgery, The First Affiliated Hospital of Jinan University, Guangzhou, China; ^2^UrinarySurgery, Southern Hospital of Southern Medical University, Guangzhou, China

## Abstract

**Background:**

Mizoribine (MZR) is widely used in Asia due to its high safety and low cost, and comparative studies of its safety and efficacy with the first-line drug mycophenolate mofetil (MMF) have been carried out. This paper aimed to compare the efficacy and safety of MZR and MMF in immunosuppressive therapy of renal transplantation by meta-analysis.

**Methods:**

We searched randomized controlled trials (RCTs) comparing MZR versus MMF for renal transplantation in PubMed, Excerpta Medica Database (EMBASE), Cochrane Library, Web of Science, WanFang Database, China National Knowledge Infrastructure (CNKI), and Chinese Biomedical Database (CBM). Articles were assessed for their risk of bias using the Cochrane Collaboration. Forest plots and funnel plots were also performed on the included articles.

**Results:**

A total of twelve studies with 1103 patients were selected in the analysis. No significant difference were observed between the MZR group and the MMF group for the rate of acute rejection (RR = 1.50, 95% CI 1.11 to 2.01, *P* = 0.008), patient survival (RR = 1.01, 95% CI 0.99 to 1.03, *P* = 0.56), graft survival (RR = 1.02, 95% CI 1.00 to 1.04, *P* = 0.12), leucopenia (RR = 0.69, 95% CI 0.44 to 1.10, *P* = 0.12), and liver damage (RR = 0.72, 95% CI 0.46 to 1.13, *P* = 0.15). The MZR group was associated with a lower risk of gastrointestinal disorder (RR = 0.28, 95% CI 0.13 to 0.62, *P* = 0.002) and cytomegalovirus infection (RR = 0.59, 95% CI 0.42 to 0.84, *P* = 0.003) but had a higher risk of hyperuricemia (RR 1.79, 95% CI 1.17 to 2.75, *P* = 0.007). No significant publication bias was observed among included studies. *Discussion*. MZR is similar to MMF in efficacy, and in terms of safety, MZR has a lower risk of gastrointestinal disorder and cytomegalovirus infection but a higher risk of hyperuricemia.

## 1. Introduction

Renal transplantation has been widely carried out all over the world, which would be the most mature solid organ transplantation technology at present [1]. With the renewal and application of new immunosuppressive drugs, the maturity of matching technology and renal transplantation technology, the incidence of short-term rejection after transplantation has been significantly reduced, and the incidence of adverse prognostic events caused by rejection has been reduced [2, 3]. When the shortage of organs cannot be overcome at present, how to ensure the longest functional survival of available organs is one of the hot issues discussed in clinical work.

The triple immunosuppressive regimen of calcineurin inhibitors (CNIs) combined with antiproliferative drugs and hormones has been widely used to prevent and treat rejection after renal transplantation [4, 5]. The application of CNI is the basis for the success of renal transplantation, but CNIs can cause many adverse reactions, which limit their long-term application in the clinical practice of organ transplantation. The combined use of antiproliferative drugs can reduce the dosage of CNIs, then reduce its renal injury, and will not increase the incidence of rejection [6]. Antiproliferative immunosuppressants mainly include mizoribine (MZR), azathioprine (AZA), and mycophenolate mofetil (MMF). AZA is rarely used in recipients after renal transplantation because of its severe hepatotoxicity and bone marrow suppression toxicity [7]. MMF is currently recommended as the first-line drug of antiproliferative drugs, but the application of MMF after renal transplantation is easy to cause gastrointestinal reactions such as diarrhea, abdominal pain, leucopenia, infection, and liver function damage. As a new immunosuppressant, MZR has been used in clinics. Its immunosuppressive mechanism is similar to MMF. It inhibits the de novo synthesis of guanosine monophosphate by competitively inhibiting hypoxanthine monophosphate nucleoside dehydrogenase and guanosine monophosphate synthase so as to inhibit the synthesis of RNA and DNA, further inhibit the proliferation and activation of *T* and B lymphocytes, and inhibit both cellular immunity and humoral immunity [8, 9].

In recent years, there have been some studies on the efficacy and safety of MZR compared with MMF in recipients after renal transplantation [10–12]. Some studies have shown that the effectiveness of high-dose MZR in antirejection treatment after renal transplantation is equivalent to MMF, which is even better than MMF in pulmonary infection, leucopenia, and gastrointestinal disorder. However, some literatures have reported that MZR has fewer adverse reactions as its immunosuppressive effect is weaker than MMF [13, 14]. In our paper, meta-analysis was used to analysis the literature of randomized controlled trials (RCTs) comparing MZR and MMF so as to evaluate the efficacy and safety of MZR and MMF in renal transplant recipients in order to provide evidence-based basis for clinical rational selection of immunosuppressants.

## 2. Methods

### 2.1. Literature Search Strategy

We performed a systematic search for relevant literature from the following databases up to April 2022 in PubMed, Excerpta Medica Database (EMBASE), Web of Science, Cochrane Library, WanFang Database, Chinese BioMedical Database (CBM), and China National Knowledge Infrastructure (CNKI). Search terms were constructed by using Boolean operator “AND” or “OR” of the following keywords: (1) mizoribine; (2) mycophenolate mofetil; (3) renal transplantation; and (4) kidney transplantation. No language restrictions were applied on searches. We attempted to identify additional studies by reviewing the reference lists to identify any studies that our search strategy may have missed.

### 2.2. Study Selection

We considered studies to be eligible for inclusion if they met the following criteria:

1. Population: patients after renal transplantation
2. Study design: randomized controlled trials (RCTs)
3. Intervention and control: researches comparing patients receive MZR and MMF
4. Outcomes: efficacy outcomes, such as acute rejection and patient survival; safety outcomes, such as leukopenia, cytomegalovirus infection, and hyperuricemia
5. Language: the publication was available in either English or Chinese

### 2.3. Data Extraction and Quality Assessment

Two authors (JChen and HLiu) collected data independently, and any different opinions between the two authors were resolved by discussions with the third author for a consensus decision. The data extracted from each article included basic information (study design, author's name and country, publication year, duration, and time of follow-up), and patient's demographic details (sample size, age, sex, and drug dosage). We used the Cochrane Risk of Bias Tool for methodological quality as all the included studies were RCTs.

### 2.4. Statistical Analysis

Meta-analysis was performed using Review Manager (version 5.4, Nordic Cochrane Centre) and STATA (version 14.0, STATA Corporation). We expressed dichotomous outcome data as risk ratios (RRs) with 95% confidence intervals (CIs) and continuous outcome data as mean differences (MDs) with 95% CIs. Heterogeneity of the data was assessed using I^2^ values. If I^2^ was <50%, we used a fixed-effect model to pool the data; otherwise, we used a random-effect model for meta-analysis. The funnel plot and Egger's test was conducted to assess the potential publication bias.

## 3. Results

### 3.1. Search Process

**Figure 1:**
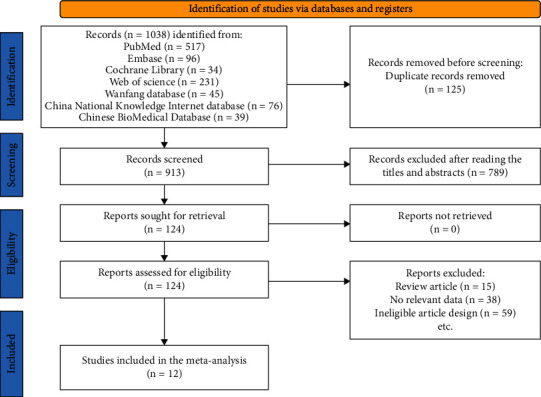
Flowchart of the literature search and study selection.

A total of 1038 potentially eligible studies were identified. Of the identified articles, 125 were duplicates and removed, 789 articles were excluded after reading the titles and abstracts. After the full-texts screening, 12 RCTs, including 1103 patients, met the inclusion criteria and were then included in this meta-analysis [15–26]. The details of our literature search and selection process are shown in Figure 1.

### 3.2. Characteristics of the Included Studies

The baseline characteristics of the selected studies are presented in Table 1. In total, 1103 patients were included. All 12 articles were published from 2003 to 2020, six came from China, five came from Japan, and one came from Korea. Four articles were published in Chinese and the others were in English. The time of follow-up ranged from 6 to 50 months.

### 3.3. Results of Quality Assessment

**Figure 2:**
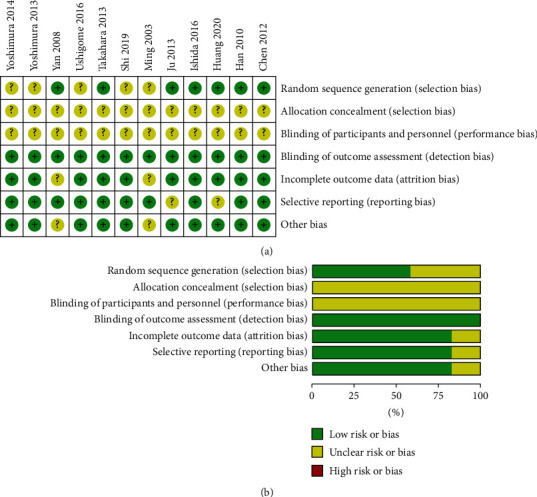
Quality assessment of included studies. (a) Risk of bias summary of each included study; (b) Overall risk of bias of included studies.

Overall, all the trials were deemed to be at unclear risk of allocation concealment (selection bias) and blinding of participants and personnel (performance bias), five studies were deemed to be at unclear risk of random sequence generation (selection bias), and all studies did not have high risk of bias (Figure 2(a)). A summary of all kind of bias in each study is shown in Figure 2(b).

### 3.4. Meta-Analysis of Efficacy Outcomes

#### 3.4.1. Acute Rejection

**Figure 3:**
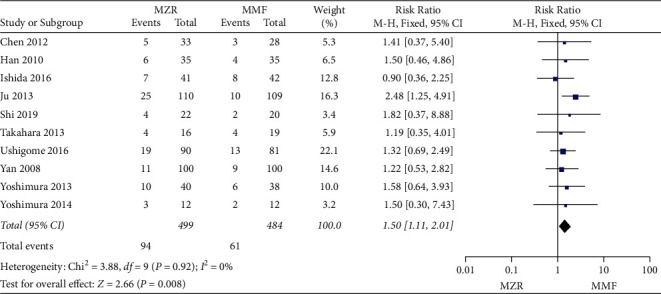
Forest plot: MZR versus MMF for acute rejection. MZR, mizoribine; MMF, mycophenolate mofetil; CI, confidence interval; df, degrees of freedom.

Ten studies comprising 983 patients provided information regarding acute rejection. The MZF group demonstrated significantly lower rate of acute rejection (RR = 1.50, 95% CI 1.11 to 2.01, *P* = 0.008, I^2^ = 0%, fixed-effect model) compared with the MMF group (Figure 3).

#### 3.4.2. Patient Survival

**Figure 4:**
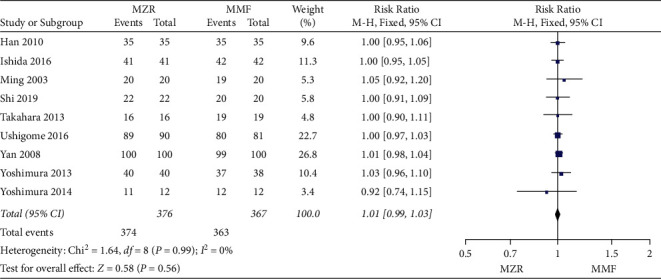
Forest plot: MZR versus MMF for patient survival. MZR, mizoribine; MMF, mycophenolate mofetil; CI, confidence interval; df, degrees of freedom.

Patient survival was reported in nine studies involving 743 patients. Pooled results failed to show statistically significant differences for patient survival between the MZR and MFF group (RR = 1.01, 95% CI 0.99 to 1.03, *P* = 0.56, I^2^ = 0%, fixed-effect model) (Figure 4).

#### 3.4.3. Graft Survival

**Figure 5:**
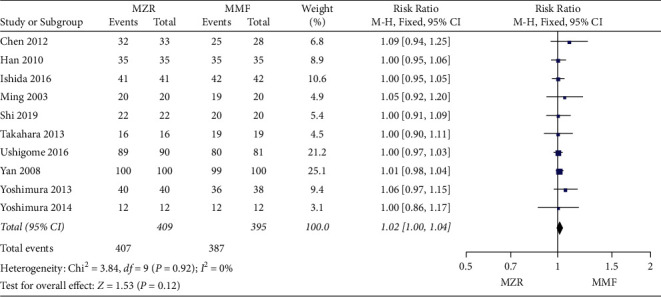
Forest plot: MZR versus MMF for graft survival. MZR, mizoribine; MMF, mycophenolate mofetil; CI, confidence interval; df, degrees of freedom.

In the evaluation of difference of graft survival between the MZR group and MMF group, ten articles involved 804 patients were included. Similarly, no statistical significance of graft survival incidence was found between the two groups (RR = 1.02, 95% CI 1.00 to 1.04, *P* = 0.12, I^2^ = 0%, fixed-effect model) (Figure 5).

### 3.5. Meta-Analysis of Safety Outcomes

#### 3.5.1. Leukopenia

**Figure 6:**
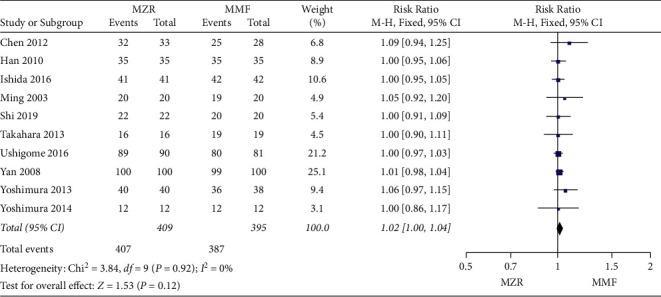
Forest plot: MZR versus MMF for leukopenia. MZR, mizoribine; MMF, mycophenolate mofetil; CI, confidence interval; df, degrees of freedom.

A total of 762 patients enrolled in nine studies were compared on the frequency of leukopenia. There was no significant difference in the incidence of leukopenia for those patients who received MZR compared with MMF (RR = 0.69, 95% CI 0.44 to 1.10, *P* = 0.12, I^2^ = 36%, fixed-effect model) (Figure 6).

#### 3.5.2. Liver Damage

**Figure 7:**
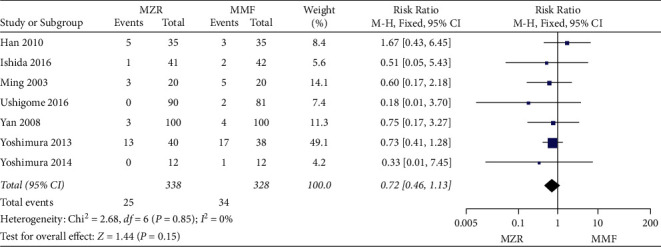
Forest plot: MZR versus MMF for liver damage. MZR, mizoribine; MMF, mycophenolate mofetil; CI, confidence interval; df, degrees of freedom.

Two studies contributed to analysis of liver damage. No significant difference in incidence of liver damage was detected in patients who were treated with MZR compared with MMF (RR = 0.72, 95% CI 0.46 to 1.13, *P* = 0.15, I^2^ = 0%, fixed-effect model) (Figure 7).

#### 3.5.3. Gastrointestinal Disorder

**Figure 8:**
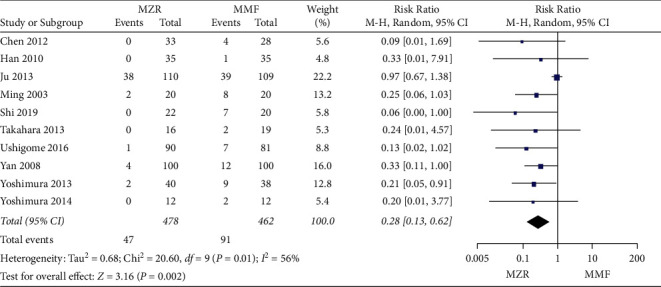
Forest plot: MZR versus MMF for gastrointestinal disorder. MZR, mizoribine; MMF, mycophenolate mofetil; CI, confidence interval; df, degrees of freedom.

Ten trials evaluated gastrointestinal disorder between the MZR group and MMF group. Significant heterogeneity was found (*P* = 0.01, I^2^ = 56%). Consequently, the random-effect model was applied. The MZR group was markedly beneficial in improving gastrointestinal disorder compared with the MMF group (RR = 0.28, 95% CI 0.13 to 0.62, *P* = 0.002) (Figure 8).

#### 3.5.4. Cytomegalovirus Infection

**Figure 9:**
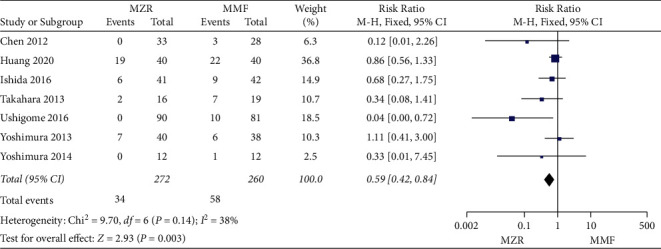
Forest plot: MZR versus MMF for cytomegalovirus infection. MZR, mizoribine; MMF, mycophenolate mofetil; CI, confidence interval; df, degrees of freedom.

With regard to cytomegalovirus infection, seven trails involving 532 patients were selected. The polled analysis showed that the MZF group had a significantly lower rate of cytomegalovirus infection than the MMF group (RR = 0.59, 95% CI 0.42 to 0.84, *P* = 0.003, I^2^ = 38%, fixed-effect model) (Figure 9).

#### 3.5.5. Hyperuricemia

**Figure 10:**
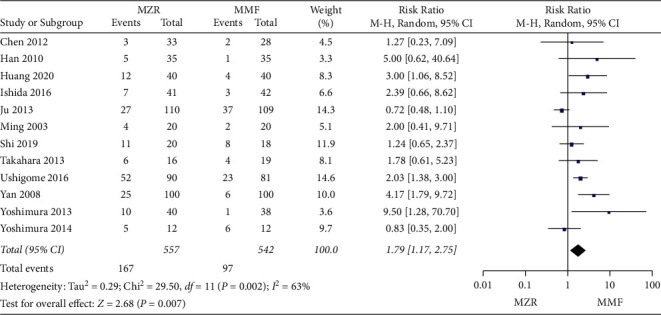
Forest plot: MZR versus MMF for hyperuricemia. MZR, mizoribine; MMF, mycophenolate mofetil; CI, confidence interval; df, degrees of freedom.

All the included studies had data available for analysis of hyperuricemia. The MZR group showed a significantly higher incidence of hyperuricemia compared with the MMF group (RR 1.79, 95% CI 1.17 to 2.75, *P* = 0.007, random-effect model). There is significant heterogeneity between the included studies (*P* = 0.002, I^2^ = 63%) (Figure 10).

### 3.6. Publication Bias

**Figure 11:**
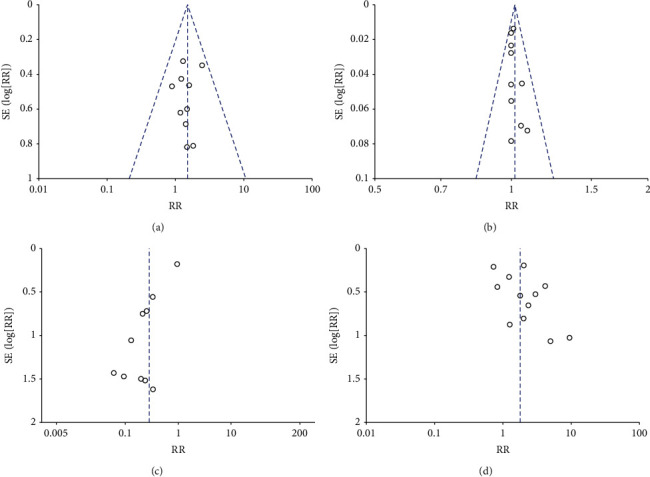
Funnel plot for publication bias in this meta-analysis (a) Acute rejection; (b) Graft survival; (c) Gastrointestinal disorder; (d) Hyperuricemia.

The publication bias test was conducted when the included studies were at least ≥10 by using the funnel plot and Egger's test, so we performed the tests on the outcomes of acute rejection, graft survival, gastrointestinal disorder, and hyperuricemia. The funnel plots for acute rejection and graft survival were visually symmetrical, and Egger's test also showed no significant publishing bias (acute rejection, *P* = 0.764; graft survival, *P* = 0.618). Even though the shape of funnel plots for gastrointestinal disorder and hyperuricemia showed some evidence of asymmetry, the *P* value of Egger's test was nonsignificant (gastrointestinal disorder, *P* = 0.185; hyperuricemia, *P* = 0.327) (Figure 11).

## 4. Discussion

At present, renal transplantation mainly relies on the classic triple immunosuppressive therapy of calmodulin phosphatase inhibitor, MMF, and hormone to control acute rejection. However, AZA has significant hepatotoxicity and hematotoxicity, MMF is often accompanied by opportunistic virus infection that is difficult to control, and it is expensive [27, 28]. Therefore, the transplantation urgently needs new drugs with a good curative effect, good safety, and moderate price so as to provide more choices for clinicians.

As an antimetabolic immunosuppressant, MZR has mild adverse reactions. According to early studies, its antirejection effect is also weaker than MMF, so it is not widely used in countries other than Japan. International reports on the application of MZR in the field of renal transplantation also come from Japan [29, 30]. It was developed as an antifungal drug in the early stage and later found to have an anticell proliferation effect. In the twenty-first century, it is usually used as an alternative drug for MMF after renal transplantation in Asia, especially in China, Japan, South Korea, and other countries [16, 19, 23]. The main reasons for choosing MZR to replace MMF are as follows:

1. MZR has an active structure similar to the antiviral drug ribavirin, so it has a certain inhibitory effect on a variety of viruses, while Japanese scholars believe that MZR may also have a certain inhibitory effect on BK virus (BKV) in the diagnosis and treatment of patients with BKV urine after renal transplantation
2. Early Brennan and other scholars have verified that MMF immunosuppressive regimen is one of the risk factors of BKV reactivation
3. The immunosuppressive effect of low-dose (1-3 mg/kg/d) MZR after renal transplantation is weaker than that of MMF, while high-dose (5-6 mg/kg/d) MZR is considered to provide the same immunosuppressive intensity as MMF

Therefore, in theory, when MMF is converted to MZR, it can not only rely on its anti-BKV activity but also increase the self-specific immune effect against BKV due to the decrease of immunosuppression so as to comprehensively inhibit the replication of BKV [31–33].

In our paper, the meta-analysis was used to evaluate the efficacy and safety of MZR and MMF in renal transplant recipients. The results showed that there was no significant difference in the incidence of acute rejection, patient survival, and graft survival rate between MZR and MMF groups, which were consistent with the results of Xing et al.[34]. In terms of safety, there was no significant difference in the incidence of leucopenia and liver damage between the MZR group and MMF group, but the incidence of gastrointestinal disorder and cytomegalovirus infection in the MZR group was lower than that in the MMF group, while the incidence of hyperuricemia was higher than that in the MMF group. Except that the difference in the incidence of cytomegalovirus infection was inconsistent with the research results of Li et al. [35], other safety results were consistent, and it may be related to the fact that Li's study only included four literatures for cytomegalovirus infection, while we included seven, and the result was more reliable.

The good tolerance of MZR in the gastrointestinal tract has obvious advantages. It can be used as an alternative treatment for diarrhea in renal transplant recipients so as to improve the compliance of renal transplant recipients. Infection is one of the main complications after renal transplantation, and it is also an important factor affecting the survival rate of recipients and transplanted kidneys, especially cytomegalovirus infection [36]. Mild cases are asymptomatic viremia, and severe cases are often life-threatening. MZR has been proved to inhibit cytomegalovirus in vitro in a dose-response relationship [37]. Its antiviral mechanism may be similar to its chemical structure and broad-spectrum antiviral drug ribavirin. MZR can reduce the incidence of infection without increasing the risk of rejection. It can effectively help renal transplant recipients through the high-risk infection period especially for high-risk infection recipients such as perioperative lung infection, retransplantation, and the use of polyclonal antibodies.

Hyperuricemia is a common adverse reaction of MZR. It mainly leads to the increase of guanine and xanthine nucleoside by inhibiting the activity of hypoxanthine nucleoside phosphate dehydrogenase so as to increase xanthine and uric acid, which is positively correlated with the drug dose. Therefore, the blood uric acid level of the recipient should be monitored during the administration of MZR [38]. If necessary, the dose of MZR can be reduced or uric acid lowering drugs such as allopurinol and benzbromarone can be added to maintain the normal blood uric acid level.

There were still some limitations in this study: (1) Although 12 literatures were included, the sample size was only 1103, which was still small; (2) the follow-up time ranged from 6 to 50 months due to the small number of literatures, and it was impossible to make subgroup analysis of short-term and long-term effects; (3) the research population was limited to China, Japan, and South Korea, and there was a lack of research on other regions; and (4) the dosage of each study and the type of transplanted renal were different, which may affect the accuracy of the final conclusion.

## 5. Conclusions

In conclusion, there is no significant difference in the efficacy of rejection between MZR and MMF in the prognosis of renal transplantation. In terms of safety, there is also no significant difference between the two groups in the incidence of leucopenia and liver damage; compared with the MMF group, the incidence of gastrointestinal disorder and cytomegalovirus infection in the MZR group was lower, but the incidence of hyperuricemia was higher. Limited to the design and quality of the included study, more large samples, more regions, and longer follow-up RCTs are needed to verify the conclusion.

## Figures and Tables

**Table 1:** Trial design and baseline characteristics of the 12 trials included in the meta-analysis.

Study	Country	Language	Study design	No. of patients		Gender (M/F)		Age		Dosages		Follow-up	Duration
				MZR	MMF	MZR	MMF	MZR	MMF	MZR	MMF		
Ming 2003	China	Chinese	RCT	20	20	-	-	-	-	100 mg/d	1.5 g/d	6 months	2001
Yan 2008	China	Chinese	RCT	100	100	-	-	-	-	50–100 mg/d	1 ∼ 1.5 g/d	12 months	2002
Han 2010	China	Chinese	RCT	35	35	23/12	23/12	41.6 ± 10.5	42.1 ± 10.6	150–200 mg/d	1 ∼ 1.5 g/d	12 months	September 2004 to November 2005
Chen 2012	China	Chinese	RCT	33	28	21/12	18/10	40.2 ± 11.5	38.6 ± 9.9	150–200 mg/d	1 ∼ 1.5 g/d	6 months	January 2010 to October 2010
Ju 2013	Korea	English	RCT	110	109	70/40	64/45	44.6 ± 10.9	44.2 ± 11.1	100–300 mg/d	1 ∼ 2 g/d	6 months	July 2008 to January 2011
Takahara 2013	Japan	English	RCT	16	19	9/7	15/4	36.1 ± 7.2	39.7 ± 11.3	350–600 mg/d	1 ∼ 2 g/d	12 months	July 2005 to June 2007
Yoshimura 2013	Japan	English	RCT	40	38	23/17	20/18	41 ± 13	35 ± 14	6 mg/kg/d	25 mg/kg/d	24 months	October 2004 to July 2007
Yoshimura 2014	Japan	English	RCT	12	12	7/5	5/7	50 ± 10	50 ± 13	6 mg/kg/d	1 g/d	36 months	October 2007 to April 2010
Ishida 2016	Japan	English	RCT	41	42	26/15	27/15	41.7 ± 14.4	42.3 ± 12.5	700 mg/d	2 g/d	12 months	October 2008 to December 2013
Ushigome 2016	Japan	English	RCT	90	81	56/34	50/31	42.5 ± 13.5	39.2 ± 13.1	6 mg/kg/d	30 mg/kg/d	24 months	February 2006 to June 2008
Shi 2019	China	English	RCT	22	20	20/2	16/4	30.4 ± 7.7	29.4 ± 8.4	3 mg/kg/d	1.5 g/d	36 months	January 2012 to August 2014
Huang 2020	China	English	RCT	40	40	21/19	18/22	27.5 (3–57)	28.5 (6–54)	3 mg/kg/d	15 mg/kg/d	50.7 months	March 2014 to March 2017

MZR, mizoribine; MMF, mycophenolate mofetil.

## Data Availability

The datasets used and analyzed during the current study are available from the corresponding author upon reasonable request.
